# Global ischemic heart disease burden attributable to kidney dysfunction from 1990 to 2021 and projections to 2050: results from the global burden of disease study 2021

**DOI:** 10.3389/fcvm.2025.1601549

**Published:** 2025-05-23

**Authors:** Meng Xia, Yingchao Shi, Hongtao Zhu, Yanan Ji

**Affiliations:** ^1^ Faculty of Biochemical and Environmental Engineering, Baoding University, Baoding, China; ^2^ Key Laboratory of Cardiovascular Regenerative Medicine, Central China Subcenter of National Center for Cardiovascular Diseases, Henan Cardiovascular Disease Center, Zhengzhou, China; ^3^ Department of Cardiology, Danyang People's Hospital, Danyang, China

**Keywords:** ischemic heart disease, kidney dysfunction, death, disability-adjusted life years, age-standardized rates, global burden of disease

## Abstract

**Background:**

Ischemic heart disease (IHD) is the leading cause of death of non-communicable diseases globally, presenting with particularly prominent metabolic risk associated with kidney dysfunction in the middle-aged and older populations. Accordingly, the present study intended to clarify trends in IHD burden attributable to kidney dysfunction from 1990 to 2021, with projection to 2050, in the middle-aged and older populations.

**Methods:**

This study quantified the burden of IHD attributable to kidney dysfunction in middle-aged and older populations from 1990 to 2021 through deaths, disability-adjusted life years (DALYs), and age-standardized rates (ASRs) based on the estimated annual percentage change (EAPC). Autoregressive integrated moving average (ARIMA) and exponential smoothing (ES) models were adopted to predict the changing trends of IHD burden attributable to kidney dysfunction from 2022 to 2050.

**Results:**

Between 1990 and 2021, both global deaths (from 0.83 million to 1.40 million) and DALYs (from 16.2 million to 26.1 million) from IHD attributable to kidney dysfunction increased in the studied populations. Despite rising absolute numbers, age-standardized death (ASDR) and DALY rates (ASDAR) declined significantly, with EAPC of −0.54 (95% CI: −0.97 to −0.11) and −0.55 (95% CI: −0.85 to −0.25) respectively, primarily driven by regions with high and high-middle SDI. Sex-specific analyses revealed steeper declines among females (ASDR EAPC: −1.71; ASDAR EAPC: −1.55) than males (ASDR EAPC: −1.18; ASDAR EAPC: −1.09), even with consistently higher rates in males. Age-stratified data showed peak ASRs in the >95 age group in 2021, despite consistent rate reductions across all age cohorts since 1990. Projections suggested continued growth in absolute burden through 2050, accompanied by sustained declines in ASDR and ASDAR, revealing both aging and improved age-adjusted disease management over time.

**Conclusion:**

This study suggests a decline in the global age-standardized IHD (ASDR/ASDAR) attributable to kidney dysfunction over three decades, yet accompanied by substantial absolute burden, disproportionately impacting lower SDI regions, males and the elderly. Projection to 2050 highlights a rising burden, necessitating prioritized resource allocation, enhanced health literacy, and evidence-based prevention targeting high-risk populations.

## Introduction

Ischemic heart disease (IHD) is the primary cause of mortality of non-communicable diseases globally and cardiovascular-related fatality (1). IHD stems from myocardial damage resulting from imbalanced coronary blood flow and myocardial demand, triggering by altered coronary circulation. Moreover, post-myocardial ischemia restoration of blood flow may give rise to reperfusion injury, oxidative stress, inflammatory responses and mitochondrial dysfunction, further aggravating myocardial injury (2).

According to the latest Global Burden of Disease (GBD) data, IHD caused approximately 8.99 million deaths and 180 million disability-adjusted life years (DALYs) globally, second only to COVID-19 in 2021 (3). By 2050, global deaths and DALYs attributable to IHD are projected to reach 16 million and 302 million, respectively, representing an 80% increase in deaths and a 62% increase in DALYs compared with those in 2021 (4). Therefore, to build a strong global public health system, the great challenge lies in addressing IHD through a comprehensive prevention and control strategy.

Metabolic syndrome refers to a group of risk factors, including obesity, diabetes, and hypertension, that occur together and markedly increase the risk of IHD. The burden of IHD has been increased with the aging of the worldwide population (5–7). Kidney dysfunction stands out as a powerful independent risk factor that leads to significantly elevated incidence of premature mortality and heavier burden of cardiovascular disease (8, 9). Meanwhile, in predicting the occurrence of fatal and nonfatal cardiovascular events, proteinuria and a reduction in the estimated glomerular filtration rate (eGFR) of kidney dysfunction exceed the combined effect of other traditional cardiovascular risk factors in the clinical setting (10, 11). Hence, it is crucial to diagnose kidney dysfunction in patients with IHD at an earlier stage and adopt proper treatments, aiming at controlling disease progression, lowering the risk of adverse cardiovascular events, and ultimately enhancing both patient survival and quality of life. According to the World Health Organization, there are around 5 to 10 million deaths attributable to kidney dysfunction each year, and over 4 million cases are expected to be diagnosed by 2036 (12). The incidence of kidney dysfunction increases significantly with age, especially in middle-aged and older populations. Moreover, lifestyle-related diseases such as hypertension and diabetes are major risk factors for kidney dysfunction (13). In the context of serious aging, it is critical to evaluate the temporal trends of the global IHD burden attributable to kidney dysfunction effectively. It may eventually facilitate the implementation of targeted prevention and intervention strategies, prioritization of health resources, and development of efficient health policies.

Here, by collecting data from the GBD 2021, this study estimated the burden of IHD attributable to kidney dysfunction in middle-aged and older populations between 1990 and 2021 quantified by deaths, DALYs and age-standardized rates (ASRs), including the age-standardized death rate (ASDR), the age-standardized years lived with disability rate (ASYR), the age-standardized years of life lost (ASLR), and the age-standardized disability-adjusted life years rate (ASDAR). Meanwhile, autoregressive integrated moving average (ARIMA) and exponential smoothing (ES) models were used to forecast the changing trends of IHD burden attributable to kidney dysfunction from 2022 to 2050.

## Methods

### Data sources

The GBD 2021 database (https://ghdx.healthdata.org/gbd-2021) meticulously assesses the ramifications of 371 distinct diseases, 88 risk factors, and various injuries (3) within five sociodemographic index (SDI) categories, encompassing 204 countries and territories. This database offers continuously refined estimates pertaining to the exposure of risk factors, the concomitant health risks, and the quantifiable fraction of disease burden that can be attributable to specific diseases or injuries associated with particular risk factors (14). These estimates are based on statistical models and associative analyses at the group level, thereby precluding the direct inference of causal relationships. Therefore, this study was performed based on the extraction of data from GBD 2021 database to analyze death, DALYs, years of life lost (YLLs), and years lived with disability (YLDs) due to IHD attributable to kidney dysfunction.

### Burden indicators

To delineate the temporal trends in the burden of IHD attributable to kidney dysfunction between 1990 and 2021, this study employed four pivotal metrics of the ASDR, ASYR, ASLR, and the ASDAR. The ASR per 100,000 individuals was computed via the following equation:

$$\mathrm{ASR}=\frac{\sum_{i=1}^{A}\;a_{i}w_{i}}{\sum_{i=1}^{A}\;w_{i}}\times 100,000$$

where $a_{i}$ represents the age-specific rates in the ith age group, and $w_{i}$ denotes the reference standard population weights (or the number of the standard population) for the ith age group. The estimated annual percentage change (EAPC) was further employed to analyze trends in ASR over a specified time interval, which is calculated as follows:

$$\mathrm{EAPC}=(e^{\beta }-1)\times 100\text{\%}$$

where β is the slope derived from the linear regression of the natural logarithm of the ASR during the year.

### Forecasting models

Forecasting the burden of ischemic heart disease attributable to kidney dysfunction until 2050 involved the application of ARIMA and ES models to analyze historical data. The ARIMA model is adept at capturing trends and seasonality in data by adjusting the parameters (*p*, *d*, *q*), where *p* represents the autoregressive term, *d* indicates the number of differencing operations required for achieving stationarity, and *q* signifies the number of moving average terms utilized. Candidate ranges of *p* and *q* are identified preliminarily by analyzing auto-correlation function (ACF) and partial auto-correlation function (PACF). The selection of optimal *p* and *q* values is guided by evaluating the akaike information criterion (AIC) and bayesian information criterion (BIC). The residual sequence values in the ACF/PACF graphs falling into the confidence interval and passing the Ljung-box test (*p*-value > 0.05) suggested that the residual sequence is classified as white noise sequence. Additionally, assessing the training set's performance using the root mean square error (RMSE), mean absolute error (MAE) and mean absolute percentage error (MAPE) provided insight into the model's predictive accuracy. The ES model focuses on recent observations to project potential future scenarios. It employs exponential smoothing, weighting forecasts by historical data where newer data carries more weight than older data. The optimal model was determined by the AIC/BIC, RMSE, MAE, MAPE and passing the evaluation of residual randomness.

### Statistical analyses

All data were analyzed via R software (version 4.1.0), with a threshold *P* value < 0.05 denoting statistical significance. The 95% uncertainty intervals (UIs) were calculated on the basis of the 2.5th and 97.5th percentiles derived from 1,000 iterations of the posterior distribution at each estimation phase. The confidence interval (CI) for the EAPC was determined through a linear regression model.

## Results

### The global burden of IHD attributable to kidney dysfunction

In 2021, the number of deaths caused by IHD attributable to kidney dysfunction reached 1,398,574 (95% UI: 976,437–1,778,399), indicating a sharp increase compared with the 828,212 (95% UI: 594,880–1,050,788) cases documented in 1990 (Table 1). Meanwhile, the number of DALYs associated with IHD attributable to kidney dysfunction increased to 26,134,286 (95% UI: 18,902,485–33,382,272) in 2021, whereas 16,229,802 (95% UI: 11,947,344–20,312,854) cases were reported in 1990 (Table 2).

**Table 1:** The death and ASDR for ischemic heart disease attributable to impaired kidney function from 1990 to 2021.

Characteristics	1990		2021		EAPC (95% CI)
	Number	ASR (per 100,000)	Number	ASR (per 100,000)	
Global	828,212 (594,880–10,50,788)	26.53 (18.85–33.72)	13,98,574 (976,437–17,78,399)	17.18 (11.94–21.83)	−0.54 (−0.97–0.11)
Sex					
Female	429,107 (301,908–545,491)	23.72 (16.79–30.28)	671,640 (459,634–857,556)	14.27 (9.76–18.2)	−1.71 (−1.76–1.67)
Male	399,105 (290,360–503,645)	29.82 (21.55–37.69)	726,934 (518,163–929,944)	20.78 (14.76–26.5)	−1.18 (−1.21–1.14)
SDI					
High SDI	283,180 (198,594–364,807)	26.23 (18.51–33.77)	247,958 (174,436–319,419)	9.68 (6.86–12.47)	−3.48 (−3.6–3.36)
High-middle SDI	242,140 (169,421–308,978)	31.87 (22.51–40.57)	369,622 (252,988–480,879)	19.5 (13.41–25.5)	−1.74 (−1.93–1.54)
Middle SDI	159,277 (116,144–201,595)	21.62 (15.52–27.31)	427,797 (297,227–550,315)	18.81 (12.9–24.18)	−0.26 (−0.36–0.16)
Low-middle SDI	110,261 (78,770–141,436)	22.52 (16.13–29.05)	281,111 (201,825–360,775)	23.18 (16.33–29.63)	0.25 (0.16–0.33)
Low SDI	32,150 (22,882–42,292)	18.46 (13.04–24.13)	70,676 (49,139–91,608)	18.32 (12.75–23.63)	0.13 (−0.01–0.26)
Age					
40–44 years	11,296 (7,886–15,503)	3.94 (2.75–5.41)	17,466 (11,865–24,208)	3.49 (2.37–4.84)	−0.67 (−0.76–0.57)
45–49 years	16,538 (11,907–22,310)	7.12 (5.13–9.61)	27,197 (19,716–37,410)	5.74 (4.16–7.9)	−0.79 (−0.87–0.71)
50–54 years	27,444 (19,899–36,904)	12.91 (9.36–17.36)	42,609 (30,111–57,896)	9.58 (6.77–13.01)	−0.97 (−1.07–0.88)
55–59 years	40,171 (28,652–54,208)	21.69 (15.47–29.27)	65,040 (45,080–89,858)	16.44 (11.39–22.71)	−0.93 (−1.04–0.82)
60–64 years	61,683 (44,147–81,290)	38.41 (27.49–50.61)	90,197 (64,284–120,276)	28.18 (20.09–37.58)	−1.23 (−1.33–1.13)
65–69 years	82,011 (59,988–105,549)	66.35 (48.53–85.39)	128,083 (91,568–167,401)	46.43 (33.2–60.69)	−1.44 (−1.58–1.31)
70–74 years	100,527 (71,185–129,592)	118.74 (84.08–153.07)	166,423 (114,904–215,885)	80.85 (55.82–104.88)	−1.44 (−1.58–1.3)
75–79 years	137,252 (93,626–179,799)	222.97 (152.1–292.09)	179,766 (122,409–238,251)	136.31 (92.82–180.65)	−1.42 (−1.52–1.32)
80–84 years	134,030 (84,175–179,999)	378.87 (237.95–508.82)	212,356 (132,807–292,511)	242.46 (151.64–333.98)	−1.44 (−1.55–1.34)
85–89 years	112,899 (75,116–148,832)	747.13 (497.09–984.92)	210,992 (140,677–282,388)	461.47 (307.68–617.62)	−1.54 (−1.61–1.47)
90–94 years	62,683 (43,300–82,160)	1,462.79 (1,010.45–1,917.31)	158,699 (108,957–208,698)	887.11 (609.06–1,166.61)	−1.65 (−1.72–1.59)
95 + years	27,436 (19,027–36,006)	2,694.86 (1,868.88–3,536.68)	80,413 (55,681–106,789)	1,475.38 (1,021.62–1,959.32)	−2.07 (−2.18–1.96)

**Table 2:** The DALYs and ASDAR for ischemic heart disease attributable to impaired kidney function from 1990 to 2021.

Characteristics	1990		2021		EAPC (95% CI)
	Number	ASR (per 100,000)	Number	ASR (per 100,000)	
Global	16,22,9802 (11,94,7344–20,31,2854)	450.95 (328.61–568.32)	26,13,4286 (18,90,2485–33,38,2272)	309.84 (222.64–395.39)	−0.55 (−0.85–0.25)
Sex					
Female	73,48,051 (52,87,828–92,57,761)	372.46 (268.11–468.62)	10,99,2975 (76,60,580–13,97,6956)	236.85 (165.45–300.8)	−1.55 (−1.6–1.51)
Male	88,81,750 (65,15,790–11,24,2001)	537.77 (394.9–677.85)	15,14,1311 (10,94,5668–19,59,1372)	391.58 (285.64–503.84)	−1.09 (−1.12–1.05)
SDI					
High SDI	44,92,532 (32,39,142–56,75,926)	408.18 (295.18–515.7)	35,55,959 (25,46,007–45,33,696)	156.04 (112.49–196.26)	−3.36 (−3.5–3.22)
High-middle SDI	45,09,444 (32,31,148–57,14,455)	513.46 (366.68–645.46)	60,02,845 (42,17,730–78,10,465)	310.47 (219.16–403.75)	−1.86 (−2.11–1.61)
Low-middle SDI	27,53,568 (19,90,737–35,56,002)	462.16 (331.59–590.84)	63,89,959 (46,07,035–82,02,054)	457.91 (331.56–585.79)	0.09 (0.02–0.15)
Middle SDI	36,49,435 (26,73,847–46,45,355)	390.74 (285.55–492.98)	85,04,374 (61,13,640–11,00,7058)	335.79 (240.63–432.57)	−0.37 (−0.44–0.29)
Low SDI	802,165 (571,994–10,55,119)	373.32 (266.6–489.32)	16,56,537 (11,49,341–21,35,341)	348.43 (242.95–450.84)	−0.19 (−0.28–0.09)
Age					
40–44 years	546,225 (381,902–749,311)	190.67 (133.31–261.56)	846,377 (575,395–11,75,473)	169.19 (115.02–234.98)	−0.66 (−0.75–0.56)
45–49 years	717,559 (516,937–969,283)	309.03 (222.63–417.44)	11,86,540 (861,188–16,41,324)	250.59 (181.88–346.63)	−0.78 (−0.86–0.7)
50–54 years	10,60,341 (770,202–14,23,594)	498.82 (362.33–669.7)	16,54,144 (11,69,543–22,50,248)	371.78 (262.86–505.76)	−0.96 (−1.05–0.86)
55–59 years	13,62,111 (970,328–18,39,853)	735.48 (523.94–993.44)	22,17,419 (15,37,749–30,70,818)	560.34 (388.59–775.99)	−0.91 (−1.02–0.81)
60–64 years	18,08,428 (12,92,750–23,87,203)	1,125.98 (804.9–1,486.34)	26,58,834 (18,89,135–35,46,938)	830.76 (590.27–1,108.25)	−1.21 (−1.31–1.11)
65–69 years	20,35,581 (14,94,794–26,11,240)	1,646.78 (1,209.29–2,112.49)	32,00,165 (22,93,121–41,86,856)	1,160.14 (831.32–1,517.84)	−1.42 (−1.54–1.29)
70–74 years	20,52,469 (14,62,259–26,42,374)	2,424.33 (1,727.19–3,121.12)	34,31,505 (23,69,665–44,67,259)	1,667.08 (1,151.22–2,170.27)	−1.41 (−1.55–1.28)
75–79 years	22,28,737 (15,31,286–29,22,886)	3,620.69 (2,487.65–4,748.37)	29,48,546 (19,95,646–39,32,343)	2,235.7 (1,513.18–2,981.66)	−1.4 (−1.5–1.3)
80–84 years	17,03,308 (10,67,347–22,88,626)	4,814.88 (3,017.16–6,469.45)	27,09,397 (16,93,820–37,28,644)	3,093.52 (1,933.96–4,257.27)	−1.43 (−1.53–1.33)
85–89 years	11,37,539 (754,916–15,01,851)	7,527.85 (4,995.78–9,938.74)	21,34,432 (14,23,556–28,51,827)	4,668.3 (3,113.52–6,237.35)	−1.53 (−1.6–1.46)
90–94 years	546,685 (376,858–716,036)	12,757.56 (8,794.44–16,709.56)	13,91,157 (953,757–18,20,845)	7,776.44 (5,331.42–10,178.36)	−1.64 (−1.7–1.57)
95 + years	2,25,244 (1,56,663–2,94,745)	22,124.21 (15,387.95–28,950.93)	6,61,433 (4,58,839–8,73,295)	12,135.69 (8,418.59–16,022.85)	−2.09 (−2.2–1.97)

**Figure 1:**
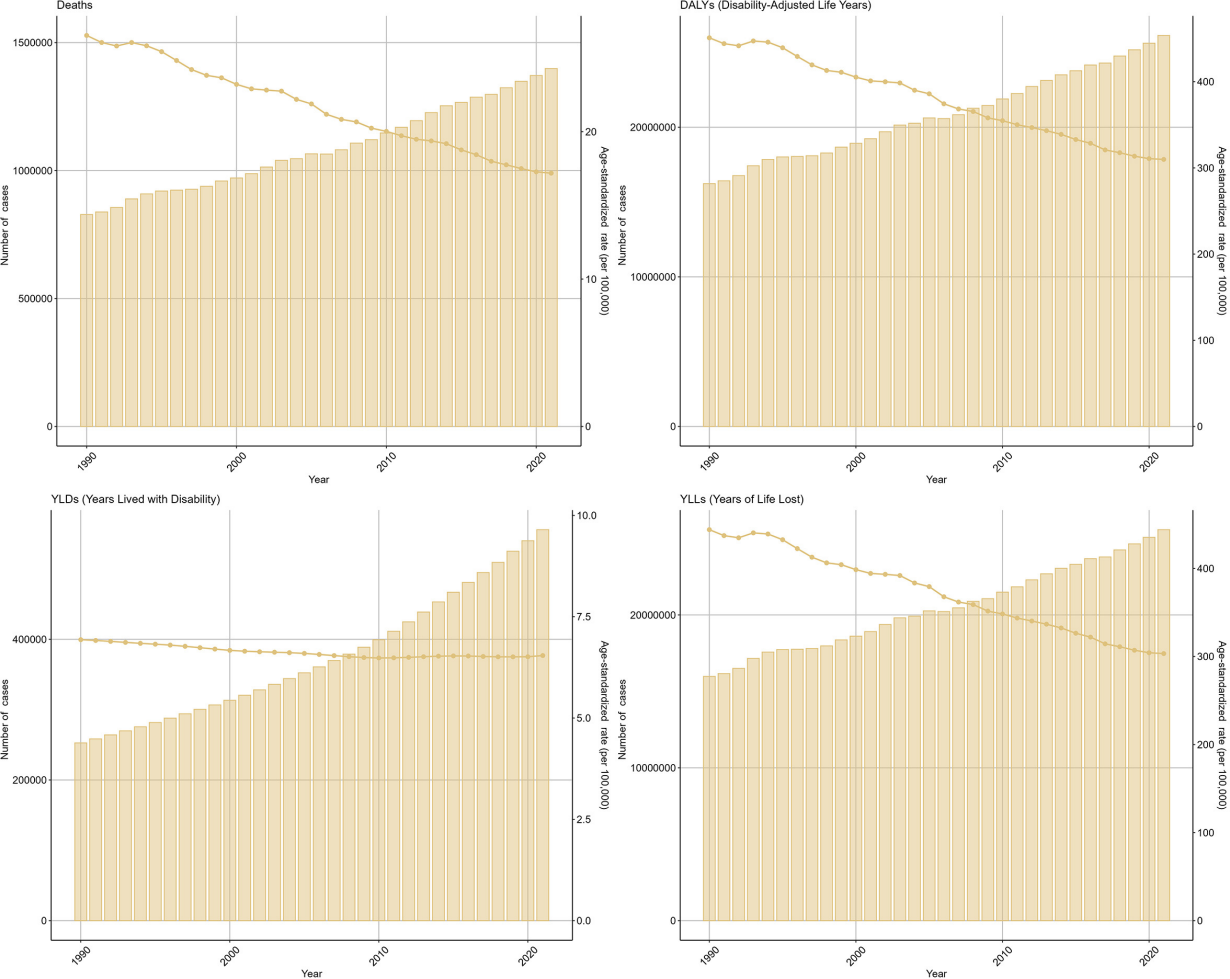
Trends in the global burden for IHD attributable to kidney dysfunction from 1990 to 2021.

The ASDR for IHD attributable to kidney dysfunction decreased from 26.53 (95% UI: 18.85–33.72) in 1990 to 17.18 (95% UI: 11.94–21.83) in 2021, with an EAPC of −0.54 (95% CI: −0.97 to −0.11). Similarly, the ASDAR declined from 450.95 (95% UI: 328.61–568.32) in 1990 to 309.84 (95% UI: 222.64–395.39) in 2021, reflecting an overall EAPC of −0.55 (95% CI: −0.85 to −0.25) (Figure 1). Consistent trends were identified in both the ASYR and the ASLR for IHD attributable to kidney dysfunction (Figure 1).

### The global trend of IHD burden attributable to kidney dysfunction at SDI levels

**Figure 2:**
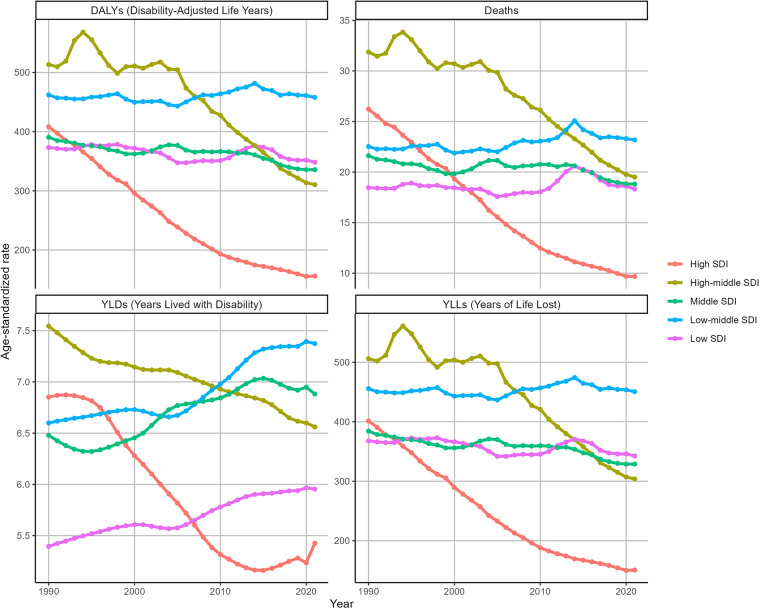
Trends in the ASRs of IHD attributable to kidney dysfunction by SDI from 1990 to 2021.

Between 1990 and 2021, there were significant reductions in ASDR and ASDAR in regions classified as High SDI and High-middle SDI (Figure 2). In High SDI regions, ASDR decreased from 26.23 (95% UI: 18.51–33.77) to 9.68 (95% UI: 6.86–12.47), with an EAPC of −3.48 (95% CI: −3.6 to −3.36). Similarly, ASDAR declined from 408.18 (95% UI: 295.18–515.7) to 156.04 (95% UI: 112.49–196.26), with an EAPC of −3.36 (95% CI: −3.5 to −3.22). In High-middle SDI regions, ASDR decreased from 31.87 (95% UI: 22.51–40.57) to 19.5 (95% UI: 13.41–25.5) (EAPC: −1.74, 95% CI: −1.93 to −1.54), while ASDAR declined from 513.46 (95% UI: 366.68–645.46) to 310.47 (95% UI: 219.16–403.75) (EAPC: −1.86, 95% CI: −2.11 to −1.61). Conversely, other SDI regions experienced marginal declines or slight increases in both ASDR and ASDAR during the same period. Figure 2 further shows the temporal variations in the YLDs and YLLs associated with IHD attributable to kidney dysfunction.

### Sex-specific burden of IHD attributable to kidney dysfunction

In 2021, the number of deaths from IHD attributable to kidney dysfunction in males [726,934 (95% UI: 518,163–929,944)] was greater than that in females [671,640 (95% UI: 459,634–857,556)], with an ASDR of 20.78 ((95% UI:14.76–26.5) for males and 14.27 ((95% UI:9.76–18.2) for females. Similar patterns were observed in the ASDAR, ASYR and ASLR for IHD attributable to kidney dysfunction (Supplementary Figure S1).

**Figure 3:**
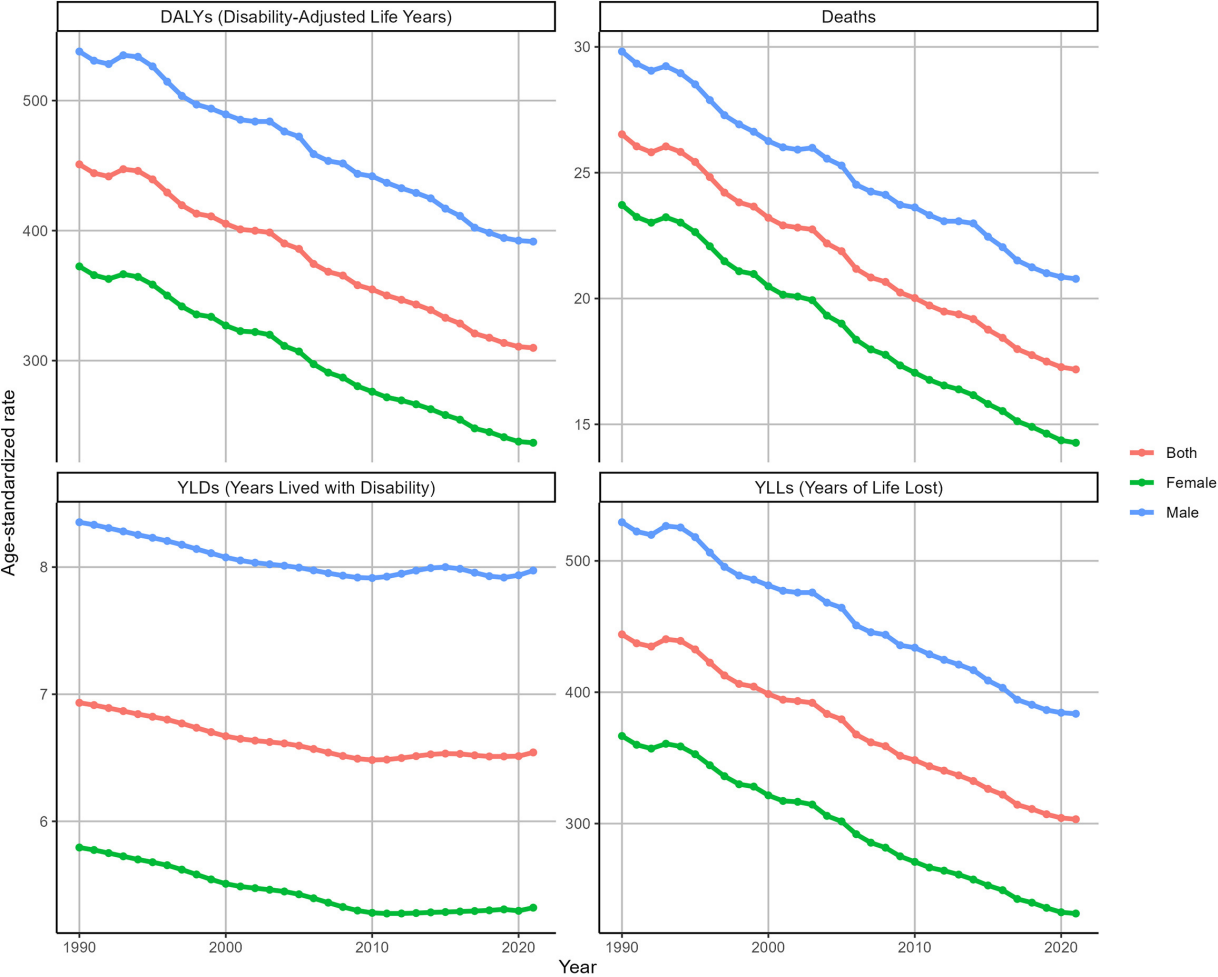
Trends in the ASRs of IHD attributable to kidney dysfunction by sex from 1990 to 2021.

From 1990 to 2021, the ASDR of IHD attributable to kidney dysfunction decreased in both females (EAPC = −1.71, 95% CI: −1.76 to −1.67) and males (EAPC = −1.18, 95% CI: −1.21 to −1.14). Similarly, the ASDAR of IHD attributable to kidney dysfunction also gradually decreased during this period for both females (EAPC = −1.55, 95% CI: −1.6 to −1.51) and males (EAPC = −1.09, 95% CI: −1.12 to −1.05). Furthermore, the ASDR and ASDAR of IHD attributable to kidney dysfunction in males were consistently greater than those in females from 1990 to 2021 (Figure 3). Consistent trends were identified in both the ASYR and the ASLR for IHD attributable to kidney dysfunction (Figure 3).

### The global burden of IHD attributable to kidney dysfunction in different age groups

**Figure 4:**
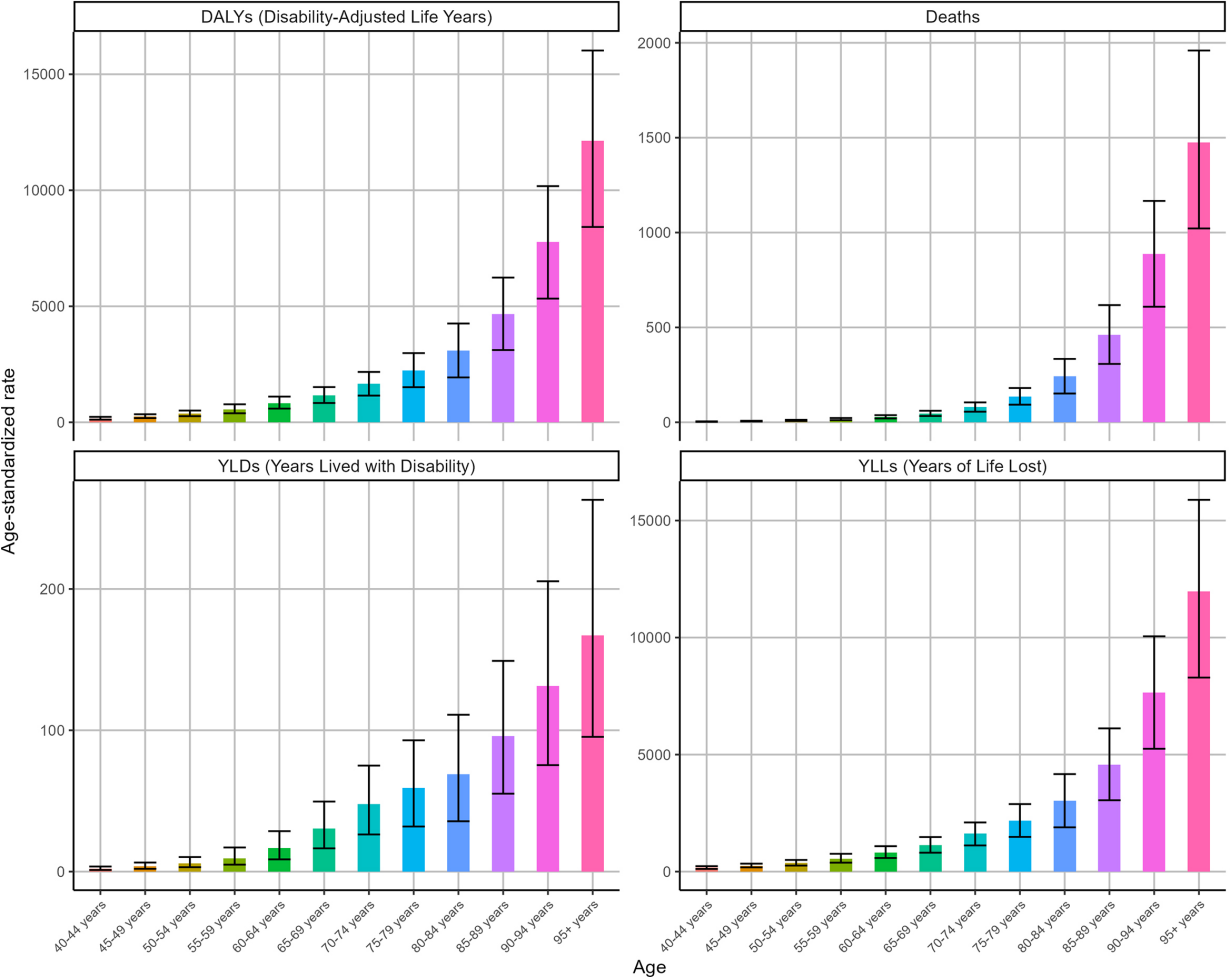
Trends in the ASRs of IHD attributable to kidney dysfunction by age in 2021.

Figure 4 shows the ASRs for IHD attributable to kidney dysfunction stratified by age in 2021. The ASDR, ASYR, ASLR, and ASDAR for IHD attributable to kidney dysfunction demonstrated remarkable consistency, all exhibiting an increase with advancing age and reaching their peak in the 95 years and older age group, with ASDR [1475.38 (95% UI: 1,021.62–1,959.32)], ASYR [167.32 (95% UI: 95.35–263.09)], ASLR [11,968.38 (95% UI: 8,290.99–15,885.38)], and ASDAR [12,135.69 (95% UI: 8,418.59–16,022.85)], respectively.

**Figure 5:**
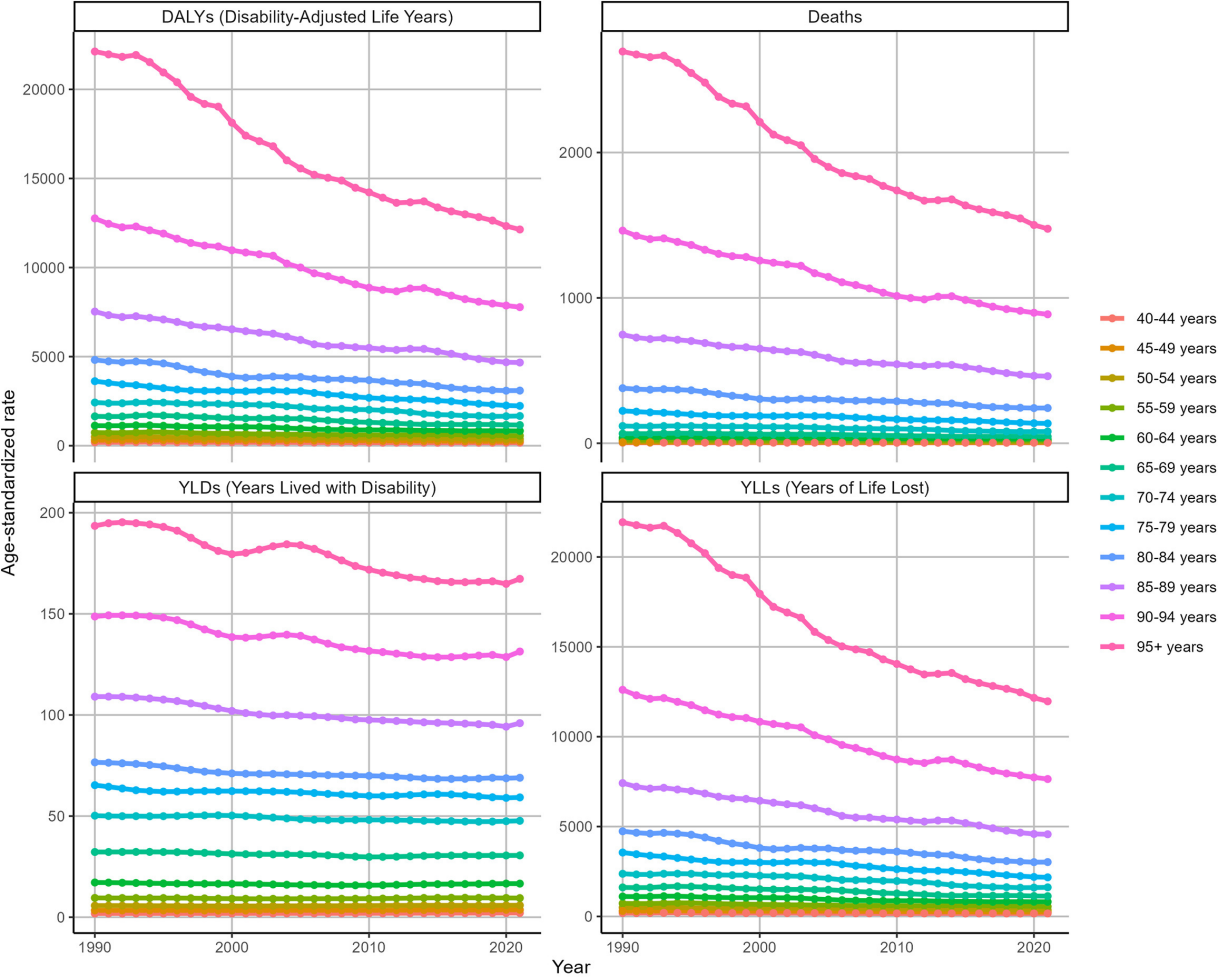
Trends in the ASRs of IHD attributable to kidney dysfunction by age from 1990 to 2021.

Overall, the ASDAR, ASDR, ASLR, and ASYR of IHD attributable to kidney dysfunction across all age groups consistently decreased during this period (Figure 5). Specifically, as the ASDR of IHD was associated with kidney dysfunction, the most rapid declines were observed in the over 95 (EAPC = −2.07, *95%* CI: −2.18 to −1.96) and 90–94 (EAPC = −1.65, 95% CI: −1.72 to −1.59) age groups. Similarly, the ASDAR of IHD attributable to kidney dysfunction among those over 95 years of age (EAPC = −2.09, 95% CI: −2.2 to −1.97) declined substantially. Figure 5 also illustrates the changes in the ASYR and ASLR associated with IHD attributable to kidney dysfunction.

### Projection of the global burden of IHD attributable to kidney dysfunction from 2022 to 2050

**Figure 6:**
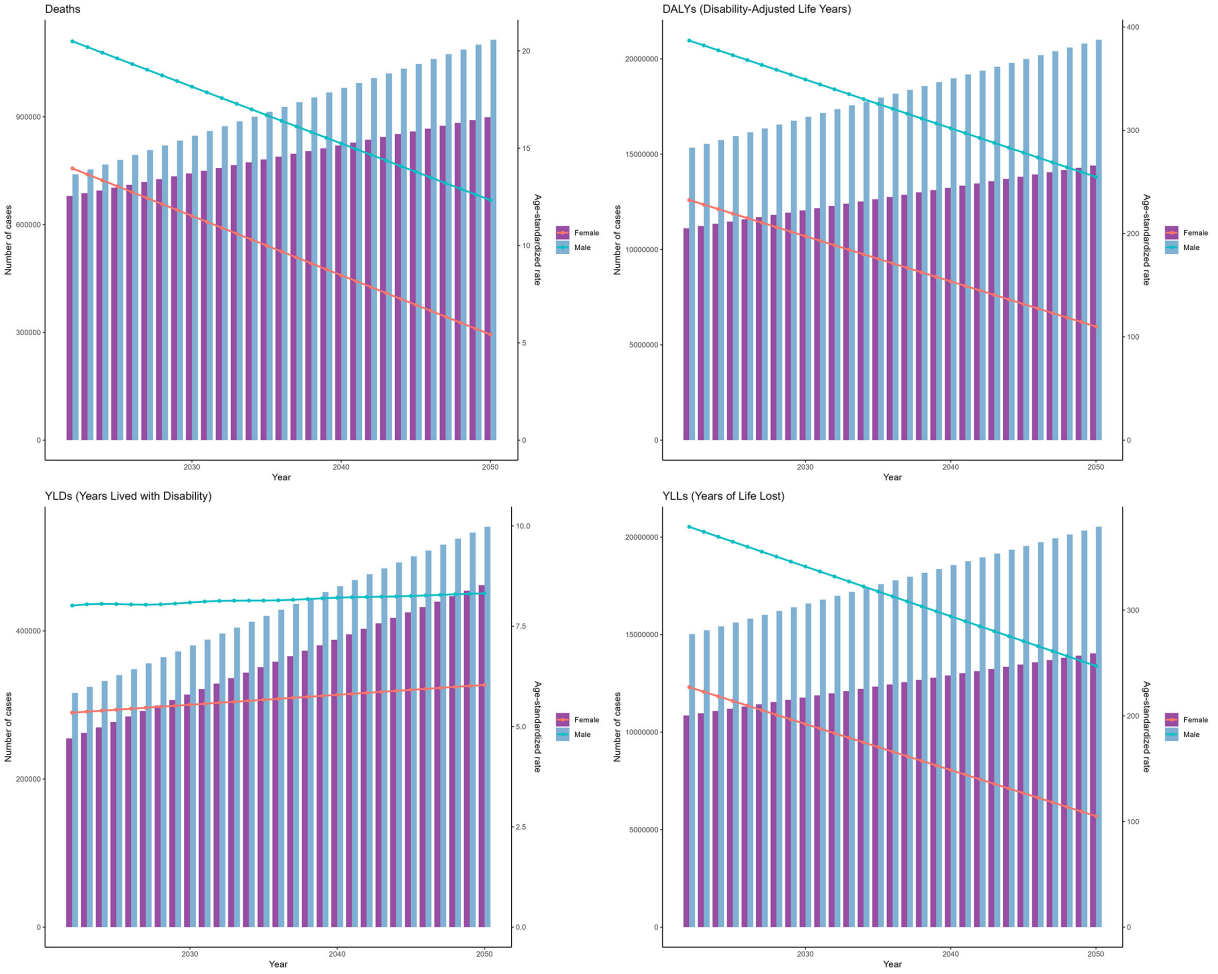
Projections of the global burden for IHD attributable to kidney dysfunction to 2050 using ARIMA model.

As predicted by the ARIMA model (Figure 6), the number of deaths from IHD attributable to kidney dysfunction for both females and males was projected to exhibit a consistently increasing trend from 2022 to 2050, reaching a peak in 2050, which were 898,526 (95% HDI: 868,028–945,168) for females and 1,114,358 (95% HDI: 982,543–1,315,950) for males, respectively. Moreover, DALYs were expected to rise gradually, reaching 14,402,743 (95% HDI: 13,900,521–15,170,825) for females and 20,997,029 (95% HDI: 20,359,880–21,971,463) for males, respectively. Figure 6 further shows the temporal variations in the YLDs and YLLs associated with IHD attributable to kidney dysfunction.

**Figure 7:**
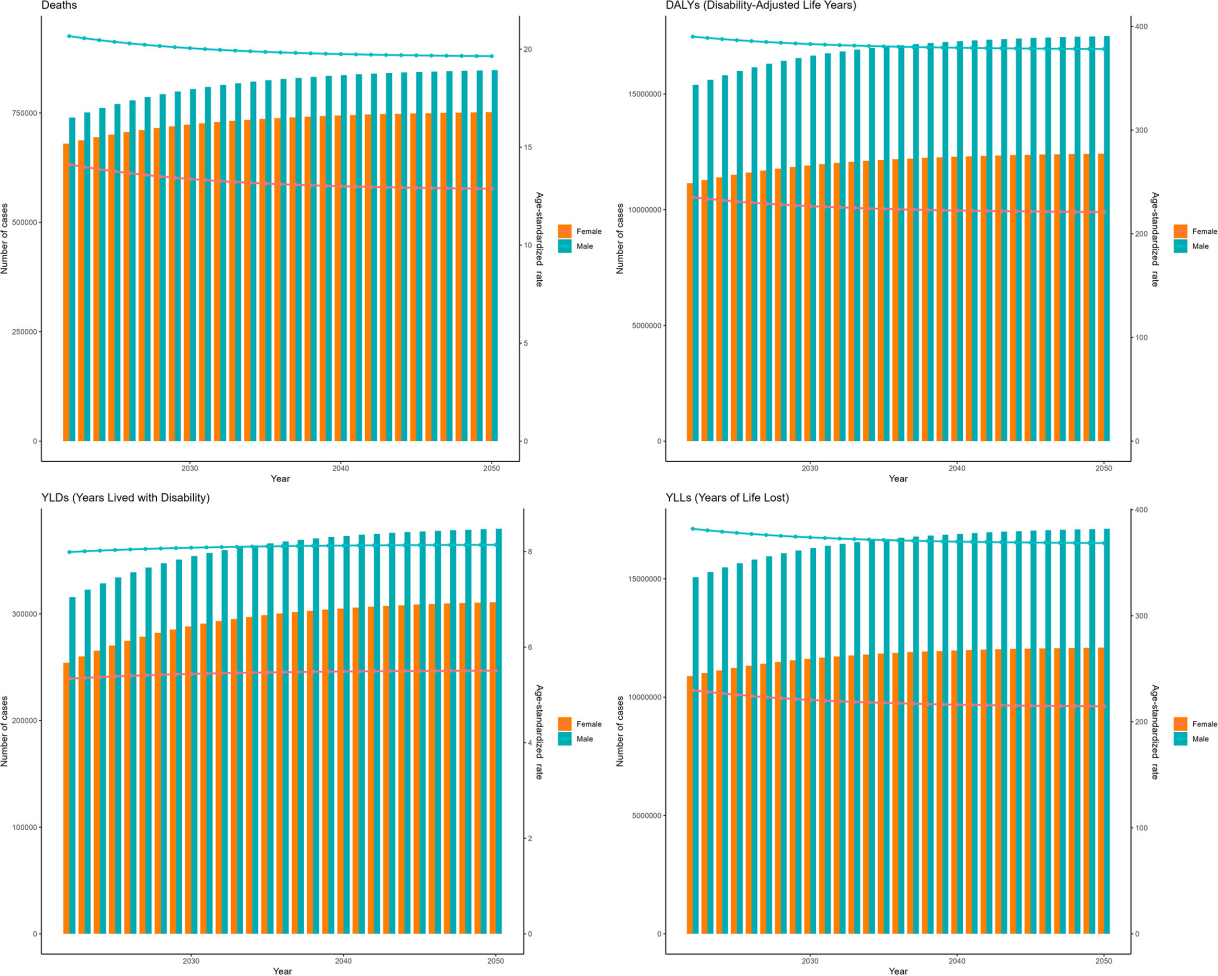
Projections of the global burden for IHD attributable to kidney dysfunction to 2050 using ES model.

Furthermore, based on the ES model (Figure 7), between 2022 and 2050, the ASDRs of IHD attributable to kidney dysfunction for both females and males were expected to gradually decrease over time, reaching their lowest values in 2050, with values of 12.87 (95% HDI: 7.03–21.81) and 19.64 (95% HDI: 13.27–29.39), respectively. The patterns of ASDAR closely mirrored those of ASDR, presenting with slow and continuous decreases by 2050 [221.00 (95% HDI: 117.99–378.55) for females, and 378.23 (95% HDI: 236.79–594.54) for males]. Figure 7 further shows the temporal variations in the YLDs and YLLs associated with IHD attributable to kidney dysfunction.

## Discussion

Our research examined the temporal evolution of IHD burden attributable to kidney dysfunction among middle-aged and elderly populations globally from 1990–2021, with a particular emphasis on sex- and age-specific epidemiological patterns. Our findings revealed a consistent global rise in the absolute burden of IHD attributable to kidney dysfunction over the specified period, despite declines in ASDR and ASDAR. This decline was predominantly driven by regions classified as High SDI and High-middle SDI. Furthermore, our analysis highlighted sex- and age-dependent enduring disparities in IHD burden distribution, underscoring a disproportionate vulnerability in male and elderly populations. Historically, males had consistently shouldered a greater burden of IHD attributable to kidney dysfunction compared to females, albeit with a gradual decrease over time. Moreover, IHD burden attributable to kidney dysfunction tended to increase with age, with ASRs peaked in the >95 age group in 2021. In addition, this study forecasted a continued escalation in the absolute disease burden by 2050 based on ARIMA and ES models.

Chronic kidney disease (CKD) is a major public health issue worldwide that is currently estimated to affect >10% of the adult population globally and significantly increases the risk of cardiovascular disease, which is the leading cause of death in this population (15). The risk of cardiovascular mortality may increase with increasing severity of CKD, which is even strongly related to mild to moderate loss of kidney function (16, 17). CKD may present with certain pathological alterations, such as heightened oxidative stress, sustained inflammatory reactions, and disturbances in mineral metabolism, all of which can expedite the progression of atherosclerosis and contribute to IHD (18–22). Furthermore, CKD patients may also experience abnormalities in calcium and phosphorus metabolism, triggering vascular calcification and hence exacerbating vascular stiffness, thereby exacerbating coronary artery stenosis and myocardial ischemia (23–25). CKD further aggravates cardiac load by increasing the volume load and the accumulation of uremic toxins; while IHD promotes the continuous deterioration of cardiac and kidney functions by intensifying kidney dysfunction, thereby forming and maintaining a harmful cycle of functional decline (26). Additionally, CKD and cardiovascular disease usually share traditional risk factors, including hypertension, diabetes, obesity, and smoking, concurrently resulting in atherosclerosis and kidney function decline, creating a detrimental cycle (27–29). In this study, during 1990–2021, due to population growth and aging, increasing trends were observed in the absolute number of deaths and DALYs, despite a downward trend in the ASDR, ASYR, ASLR and ASDAR globally, which inevitably increased the medical and economic burden on society. Early detection is a key strategy to prevent the onset, progression, and associated complications of kidney disease, but the level of awareness of kidney disease among the general population is low, as evidenced previously (30). In addition, CKD is often asymptomatic in the early stages (31–33), imposing a challenge for patients to perceive their condition, thus delaying diagnosis and treatment. Therefore, currently, the public health priorities should be increasing knowledge and implementing sustainable solutions for the early detection of kidney disease (34). Meanwhile, close monitoring and timely adjustment of treatment plans are essential for individuals diagnosed with kidney disease. Taken together, early detection and timely intervention in kidney dysfunction are anticipated to significantly alleviate the burden of IHD.

At the SDI level, High SDI and High-middle SDI regions contributed mainly to the decline in global ASRs during this period. This trend may reflect greater healthcare investments, higher public health awareness, and superior medical infrastructure. Therefore, it is imperative to adopt tailored strategies to alleviate the global disease burden. Countries with lower SDI levels should prioritize improving fundamental health infrastructure and services, advocating for healthy diets and lifestyles, and enabling early screening and intervention. Conversely, nations with higher SDI levels should emphasize enhancing healthcare systems, promoting programs for managing chronic diseases, and ensuring appropriate medication utilization.

In 2021, the global burden of IHD attributable to kidney dysfunction in middle-aged and elderly populations displayed a discernible age-dependent trend, rising in tandem with advancing age. This age-dependent disparity may be explained by age-associated functional deterioration of the kidney, markedly increasing the vulnerability to dysfunction (35, 36).

This study also reported a greater burden of IHD attributable to kidney dysfunction in men than in women in 2021. While CKD is more prevalent in women, men exhibit a more rapid deterioration in kidney function and have a greater likelihood of developing end-stage kidney disease (37–39). Consequently, it manifests as elevated rates of kidney failure and mortality in men, potentially promoting the onset of IHD and exacerbating its severity. In CKD patients, arterial and valve calcification is more severe in men than in women, which is directly correlated with a heightened risk of IHD (40). Sex hormones likely play a role in the sex disparity observed in kidney dysfunction among individuals with IHD (41). Estrogen has been shown to protect and maintain better heart and kidney function in women, while testosterone may be associated with exacerbated oxidative stress, which may stimulate the renin-angiotensin system, and worsen kidney damage in men (41–43). Traditional risk factors (e.g., hypertension, diabetes, dyslipidemia, and smoking) are common in individuals with CKD, all of which are critical in the progression of atherosclerotic cardiovascular diseases and CKD. However, sex disparity also exists in the prevalence and impact of these factors on cardiovascular disease. Men were found to exhibit a higher prevalence of hypertension compared to premenopausal women (44), and men with CKD, particularly those undergoing dialysis, demonstrated poorer ambulatory blood pressure control. At present, it is still uncertain concerning the precise etiology of this sex-dependent difference in ambulatory blood pressure regulation. However and apparently, ambulatory blood pressure levels were discovered to significantly link to CKD progression, as well as cardiovascular disease morbidity, and mortality (45, 46). Moreover, men with CKD exhibited a higher prevalence of metabolic disorders like diabetes and obesity, compounding their cardiovascular risk; in contrast, women with CKD seemed to have less likely metabolic syndrome and diabetes (47). This trend may be attributable to choices of lifestyles including smoking, alcohol consumption, and sedentary behavior (48). Meanwhile, men were surveyed to exhibit lower rates of engagement with screening and prevention programs, as well as primary care, compared to women (49, 50). Male patients with CKD often present with distinct behavioral patterns that may produce negative impacts on disease management. Firstly, men frequently delay seeking medical advice in the early stage of CKD, disregarding initial symptoms and leading to a diagnosis at an advanced stage (57). Secondly, men commonly employ risk-avoidance behaviors like smoking and excessive alcohol consumption as coping mechanisms for disease-related stress, rather than active coping. Based on an observation in predialysis CKD patients, men would encounter several challenges in crucial health care behaviors such as medication adherence, dietary control, and regular follow-up visits, thereby hastening the decline in glomerular filtration rate and accelerating disease progression (51). Furthermore, male patients are more inclined to persist in smoking and drinking, even after initiating dialysis treatment (52, 53). With respect to the above, the increased IHD burden attributable to kidney dysfunction in men is likely the result of a complex interplay of biological, clinical, and social factors. Tailored prevention and treatment strategies are indispensable for improving health outcomes in individuals with IHD attributable to kidney dysfunction, considering sex-specific disparities. Additionally, a greater IHD burden attributable to kidney dysfunction was noticed in males than in females across different age groups in 2021, as quantified by the rates of death, DALYs, YLDs and YLLs (Supplementary Figure S2), underscoring the necessity for heightened vigilance toward middle-aged and older men.

From 1990–2021, there was a consistent decrease in IHD burden attributable to kidney dysfunction in both sexes and across various age brackets. This decline hints the efficacy of global initiatives aimed at preventing IHD attributable to kidney dysfunction. Notably, the reduction in disease burden was more prominent among older age groups, probably explained by better access to standardized cardiovascular and kidney treatments, as well as early kidney function screening. Conversely, younger populations experienced a smaller or stable decline in disease burden, potentially attributable to limited health awareness and unmanaged metabolic risk factors such as hyperglycemia and hyperlipidemia (54).

Furthermore, this study carried out a comprehensive assessment utilizing ARIMA and ES models to better predict future trends of the global IHD burden attributable to kidney dysfunction and offer quantitative support for global health policy decision-making. Consistently, from 2022–2050, the overall burden would continue to rise, with persistent sex disparities, although the ASDR, ASDAR and ASLR of IHD attributable to kidney dysfunction were expected to improve. These projections underline the significant challenge of addressing IHD attributable to kidney dysfunction. Given the global trends of population growth and aging, it is imperative to allocate resources strategically toward specific demographics, while simultaneously enhancing early prevention, timely diagnosis, and efficient intervention.

Our study is subject to several limitations as well. First, this study might possess inconsistencies and gaps in data quality as it was conducted based only on GBD data, which rely on national and regional statistics for estimation. Second, variations in raw data collection among countries might result in bias considering that kidney function is calculated clinically through equations that estimate the eGFR by adjusting the serum creatine concentration in accordance with age, sex and race. Moreover, the applicability of the existing estimation equations was reported to vary possibly among different races and populations (55, 56). Additionally, our study employed ARIMA and ES models for assessment, both of which operate under the assumption that historical trends will persist in the future, disregarding unforeseen influences such as the introduction of new treatments or shifts in public health policies. Finally, findings generated from GBD data analysis, despite the generation of quantitative links between risk factors and disease burden, were observational and susceptible to confounding variables, exposure assessment inaccuracies, and model assumptions. These associations should be considered as instruments for hypothesis generation or informing public health agendas, rather than definitive proof of causation. Our study was predicated on a secondary analysis of GBD data, thus inheriting all limitations associated with the GBD dataset.

## Conclusions

This study suggests general decline in the ASDR and ASDAR of IHD attributable to kidney dysfunction in middle-aged and elderly populations globally over the past three decades, primarily driven by regions with high and high-middle SDI. Despite this trend, the overall burden remains substantial on a global scale. Sex and age disparities exist in the global burden of IHD attributable to kidney dysfunction, especially in males and elderly individuals. Projections suggest a continued increase in the absolute burden of IHD attributable to kidney dysfunction by 2050. To alleviate the global burden of IHD attributable to kidney dysfunction, there is a need for systematic implementation of strategic resource allocation prioritizing high-risk populations, enhanced health literacy and kidney dysfunction awareness campaigns, as well as evidence-based prevention with targeted mitigation measures.

## Data Availability

The original contributions presented in the study are included in the article/Supplementary Material, further inquiries can be directed to the corresponding author.

## Supplementary material

Supplementary Figure S1 The ASRs of IHD attributable to kidney dysfunction by sex in 2021.

Supplementary Figure S2 The rates of deaths, DALYs, YLDs and YLLs by sex across different age groups in 2021.
