# Supplementary figures and images for: Global ischemic heart disease burden attributable to kidney dysfunction from 1990 to 2021 and projections to 2050: results from the global burden of disease study 2021
Source: Front Cardiovasc Med. 2025 May 23;12:1601549. doi: 10.3389/fcvm.2025.1601549 (PMC12141223; doi:10.3389/fcvm.2025.1601549)

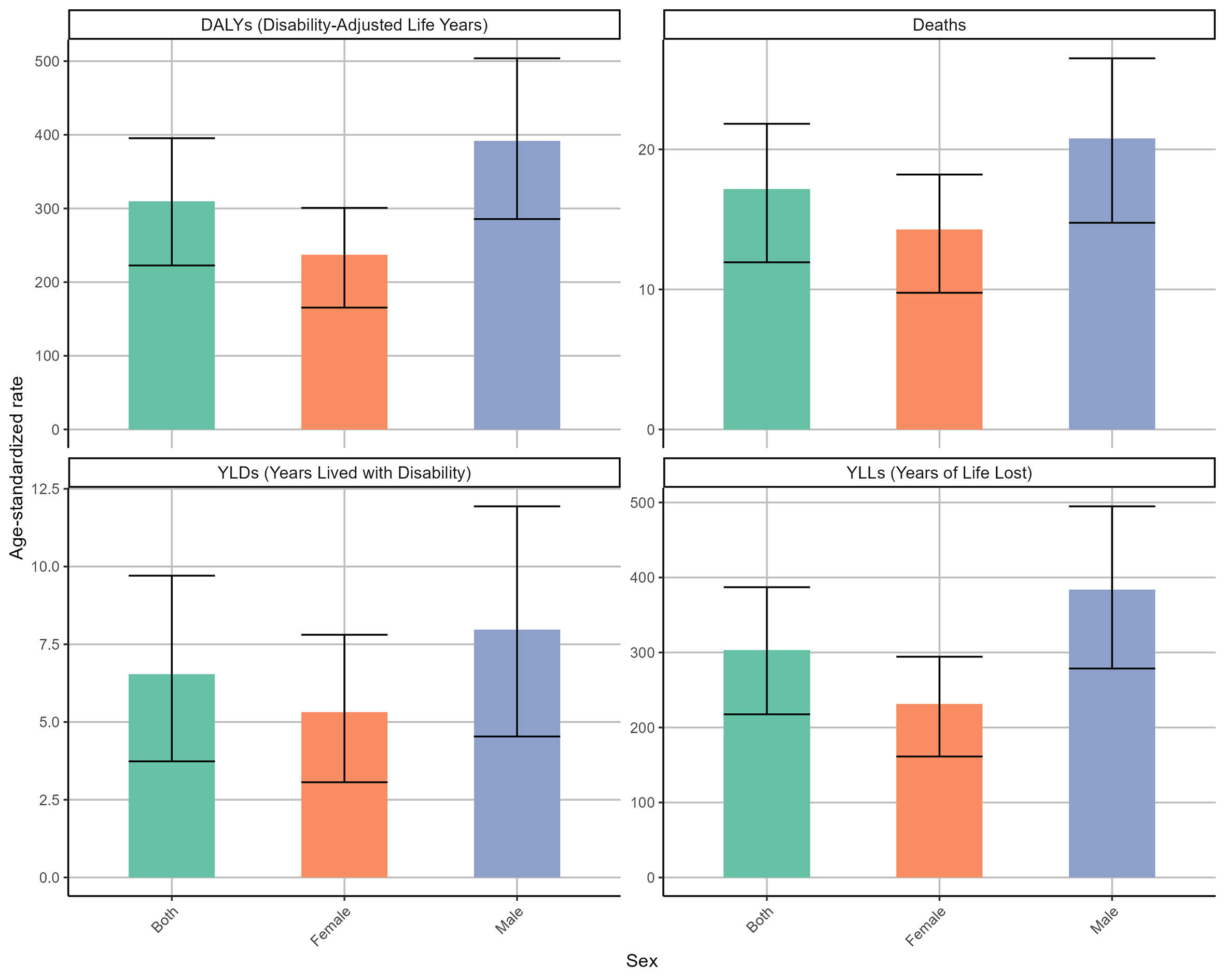

Supplement: Supplementary Figure S1 — The ASRs of IHD attributable to kidney dysfunction by sex in 2021. [file Image1.jpeg]

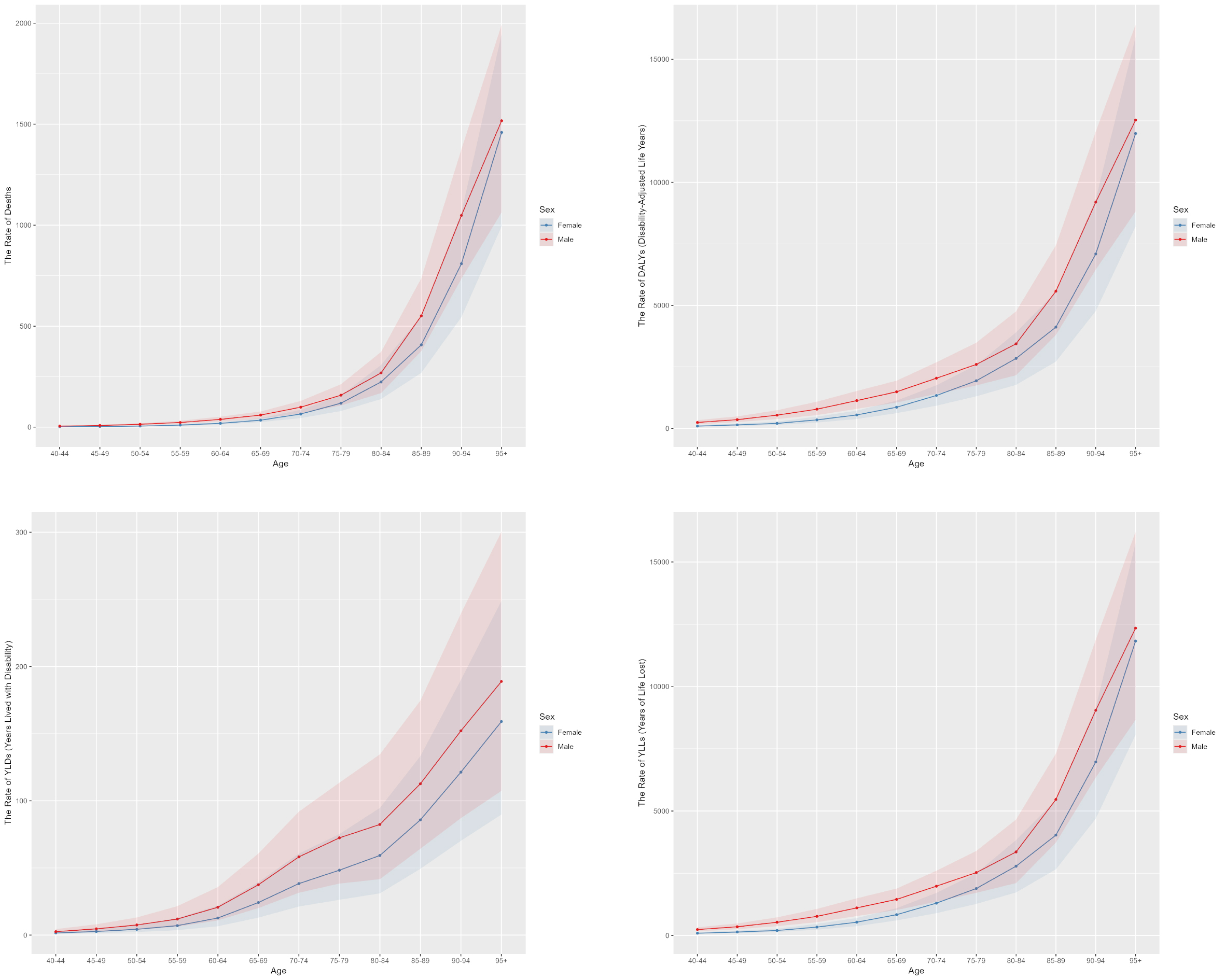

Supplement: Supplementary Figure S2 — The rates of deaths, DALYs, YLDs and YLLs by sex across different age groups in 2021. [file Image2.jpeg]
